# *Staphylococcus aureus *requires cardiolipin for survival under conditions of high salinity

**DOI:** 10.1186/1471-2180-11-13

**Published:** 2011-01-18

**Authors:** Melody Tsai, Ryosuke L Ohniwa, Yusuke Kato, Sayaka L Takeshita, Toshiko Ohta, Shinji Saito, Hideo Hayashi, Kazuya Morikawa

**Affiliations:** 1 Graduate School of Comprehensive Human Sciences, University of Tsukuba, Tennodai 1-1-1, Tsukuba 305-8575, Japan; 2 Division of Insect Sciences, National Institute of Agrobiological Sciences, Oowashi 1-2, Tsukuba 305-8634, Japan; 3 Chugoku Gakuen University, Niwase 83, Kitaku, Okayama 701-0197, Japan

## Abstract

**Background:**

The ability of staphylococci to grow in a wide range of salt concentrations is well documented. In this study, we aimed to clarify the role of cardiolipin (CL) in the adaptation of *Staphylococcus aureus *to high salinity.

**Results:**

Using an improved extraction method, the analysis of phospholipid composition suggested that CL levels increased slightly toward stationary phase, but that this was not induced by high salinity. Deletion of the two CL synthase genes, *SA1155 *(*cls1*) and *SA1891 *(*cls2*), abolished CL synthesis. The *cls2 *gene encoded the dominant CL synthase. In a *cls2 *deletion mutant, Cls1 functioned under stress conditions, including high salinity. Using these mutants, CL was shown to be unnecessary for growth in either basal or high-salt conditions, but it was critical for prolonged survival in high-salt conditions and for generation of the L-form.

**Conclusions:**

CL is not essential for *S. aureus *growth under conditions of high salinity, but is necessary for survival under prolonged high-salt stress and for the generation of L-form variants.

## Background

*Staphylococcus aureus *is an opportunistic pathogen that causes a wide range of diseases in both immunologically normal and compromised hosts. The natural habitat of *S. aureus *is the nasal cavity of warm-blooded animals. Over the past ~50 years, *S. aureus *has undergone genetic changes that have resulted in antibiotic-resistant strains [1,2]. Importantly, the methicillin-resistant strains (MRSA) are now the most common cause of nosocomial *S*. *aureus *infections and are spreading throughout communities [3].

*Staphylococcus aureus *has a number of characteristics that allow it to survive host bactericidal factors and environmental stresses, including drastic changes in osmotic pressure [4-6]. Osmoprotectants such as choline, glycine betaine, and proline accumulate in cells in response to osmotic stress [7-11]. Multiple genes, including the branched-chain amino acid transporter gene *brnQ *[12] and the arsenic operon regulatory gene *arsR *[13], cooperatively participate in salt tolerance. In addition, a very large cell wall protein, Ebh, is involved in tolerance to transient hyperosmotic pressure [14].

In general strategy, the phospholipid composition of bacteria changes in response to growth phase or environmental stressors such as osmolality [15], pH [16,17], temperature, and the presence of organic solvents [18,19]. In the 1970s, the molecular mechanism of staphylococcal salt resistance was studied, focusing on a phospholipid, cardiolipin (CL) [20]. CL possesses four acyl groups and carries two negative charges [21]. In stationary phase, 30% of the *S. aureus *cell membrane is composed of CL [22]. It has been reported that CL can stabilize liposomes during osmotic stress [23] and that it is required for the growth of *Escherichia coli *and *Bacillus subtilis *under high-salt conditions [24,25]. However, the role of CL in the molecular mechanism of staphylococcal resistance to high salinity remains unknown.

In this study, we used an improved lipid extraction method to assess the phospholipid composition of *S. aureus *and performed molecular genetic analyses to evaluate the role of CL in the resistance of *S. aureus *to high salinity.

## Results

### *Staphylococcus aureus *phospholipid composition

**Figure 1:**
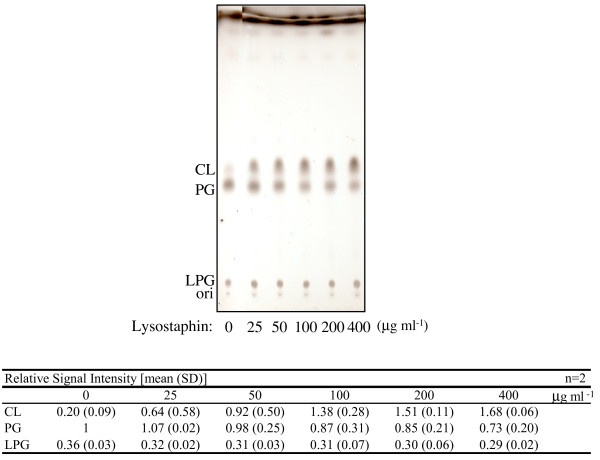
**Effect of lysostaphin treatment on CL extraction efficiency**. Prior to lipid extraction, cells (N315) were incubated for 3 min at 37°C in the presence of lysostaphin at the indicated concentrations. CL: Cardiolipin. PG: Phosphatidylglycerol. LPG: Lysyl-phosphatidylglycerol. The means and standard deviations of relative signal intensities are shown at the bottom.

The phospholipid composition of *S. aureus *grown under various conditions was analyzed. Previous studies with specific *S. aureus *strains under defined conditions have indicated that the CL level increases as the cells enter stationary phase [22] and when cultured under high-salt conditions [20]. In our initial experiments, the CL level varied among the *S. aureus *strains tested (Additional file1, Figure S1), probably because the cell wall reduced the CL extraction efficiency. Pretreatment with lysostaphin (0.1 mg ml^-1 ^for 3 min at 37°C), which degrades the Gly_5_-bridge structures in cell walls [26,27], increased the CL extraction efficiency without affecting the amounts of other phospholipids extracted (Figure 1). With this method, the CL level did not differ significantly among the strains tested (N315, NKSBm, NKSBv, MRSA No. 7, MRSA No. 33, and COL; Additional file1, Figure S1). Therefore, cells were treated with lysostaphin prior to lipid extraction in all subsequent experiments.

**Figure 2:**
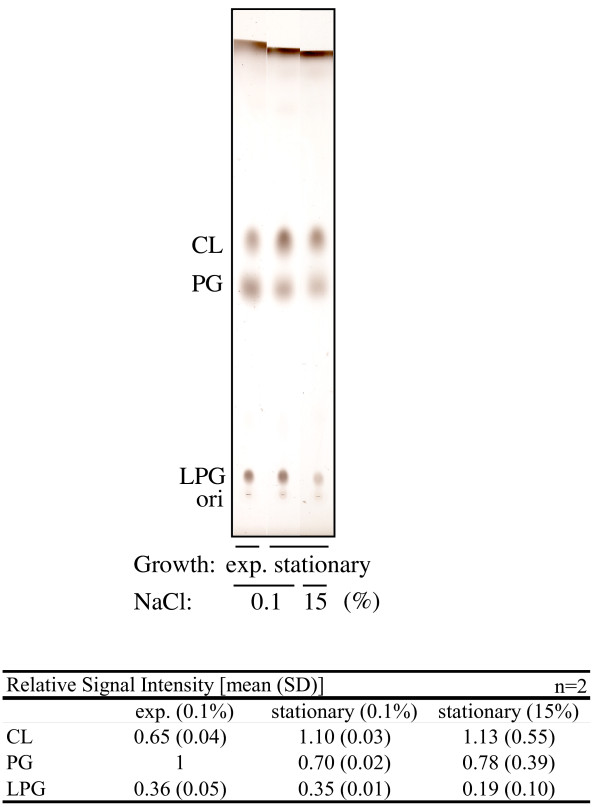
**Phospholipid composition of *S. aureus *N315 under various growth conditions**. Cells were grown in LB containing either 0.1% or 15% NaCl, and harvested during the exponential (exp.) or stationary phase. The means and standard deviations of relative signal intensities are shown at the bottom.

The phospholipid profile obtained in the present study (Figure 2) was similar to those reported by others [22,28]. The signal that intensified as cells entered stationary phase (Figs. 2 and 8) was identified as CL based on molecular mass analysis [28]. However, we did not detect a reproducible increase in the CL level in response to the addition of NaCl (Figure 2). This was the case for *S*. *aureus *N315 (Figure 2) and strain 8325-4 and its derivatives RN4220 and SH1000 (data not shown).

### Molecular genetic analysis of two cardiolipin synthase homologs

**Figure 3:**
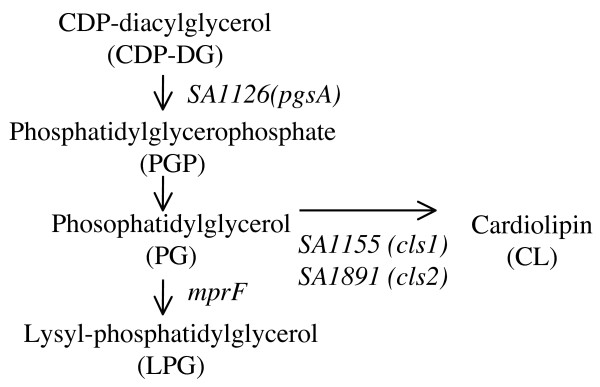
**Lipid synthesis pathway in *S. aureus *(modified from the KEGG pathway database)**. *SA1155 *(*cls1*) and *SA1891 *(*cls2*) are homologs of the *B. subtilis cls *gene.

**Figure 4:**
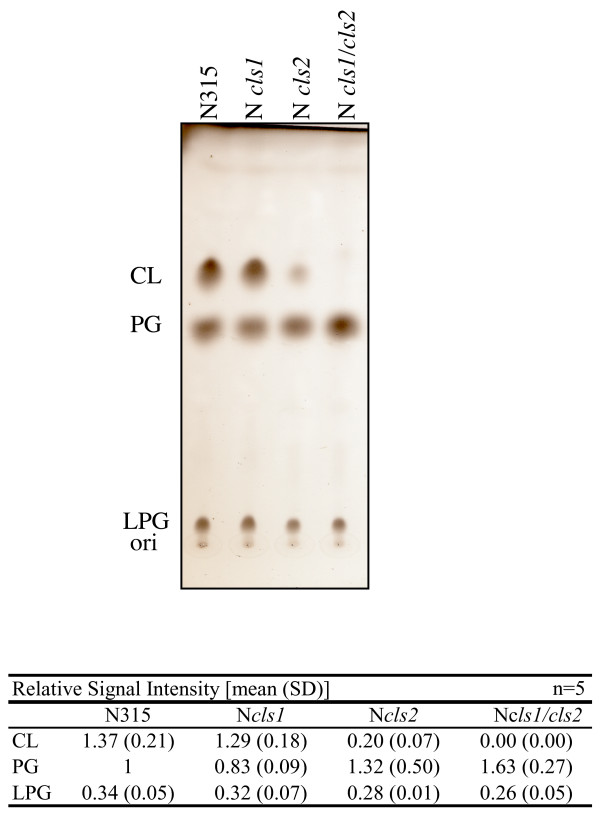
**Phospholipid composition of N315 and its isogenic *cls *mutants**. Cells were harvested during stationary phase. The means and standard deviations of relative signal intensities are shown at the bottom.

Figure 3 shows the phospholipid synthesis pathway, modified from a diagram in the KEGG pathway database [29]. A database search identified two *S. aureus *genes, *SA1155 *and *SA1891*, as homologs of *B. subtilis clsA *(CL synthase gene; one of three paralogous genes, *clsA, ywjE*, and *ywiE*) [24,30]. We constructed single and double mutants of *SA1155 *and *SA1891 *genes in *S*. *aureus *N315. No decrease in the CL level was observed in the *SA1155 *mutant (N*cls1*; Figure 4). In the *SA1891 *mutant (N*cls2*), the CL level decreased, but not completely. In the double mutant (N*cls1/cls2*), the CL signal was undetectable, and the phosphatidylglycerol (PG) signal was increased. This is consistent with the CL synthesis pathway. An identical result was observed in the mutants derived from *S*. *aureus *RN4220, 8325-4, SH1000, and MT01 (data not shown). These data strongly suggest that both *SA1155 *and *SA1891 *are CL synthase genes, and thus we refer to them as *cls1 *and *cls2*, respectively.

### Importance of CL for long-term survival under high salinity

**Figure 5:**
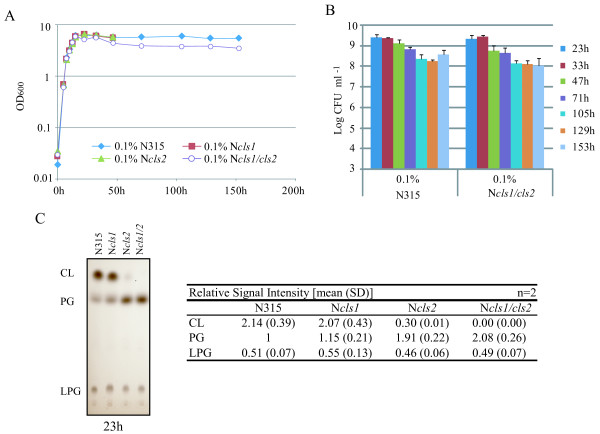
**Growth and stationary-phase survival under low salinity**. Cells were grown in 0.1% NaCl LB. **A**: Growth was monitored by optical density (OD) measurements. N315: filled diamonds; *cls1 *mutant: filled squares; *cls2 *mutant: filled triangles; *cls1*/*cls2 *double mutant: open circles. Optical densities were checked at least twice, and the means are shown. After 47 h, the OD of only N315 and its *cls1/cls2 *double mutant were measured. **B**: Number of CFUs during the long incubation. The means and standard deviations of at least three independent experiments are shown. **C**: Thin-layer chromatography of phospholipids. Cells were harvested at 23 h. The phospholipid profile was confirmed to be similar up to 153 h (data not shown). The means and standard deviations of relative signal intensities from two independent experiments are shown on the right.

Given that CL plays a regulatory role during cell replication and division in *E. coli *[28,31,32], we investigated the role of the *cls *genes in cells during growth phase transitions in 0.1% NaCl LB (Figure 5). Mutation of the *cls *genes did not affect the growth curve until 47 h (Figure 5A), after which the *cls1/cls2 *double mutant showed slightly lower optical density. However, stationary-phase CFU numbers did not differ significantly between the *cls1/cls2 *double mutant and the parent strain (Figure 5B). Moreover, the CFU numbers were sustained in both strains until at least 700 h post-inoculation (data not shown). We conclude that CL is not necessary for cell growth and stationary phase survival of *S. aureus *under these conditions.

**Figure 6:**
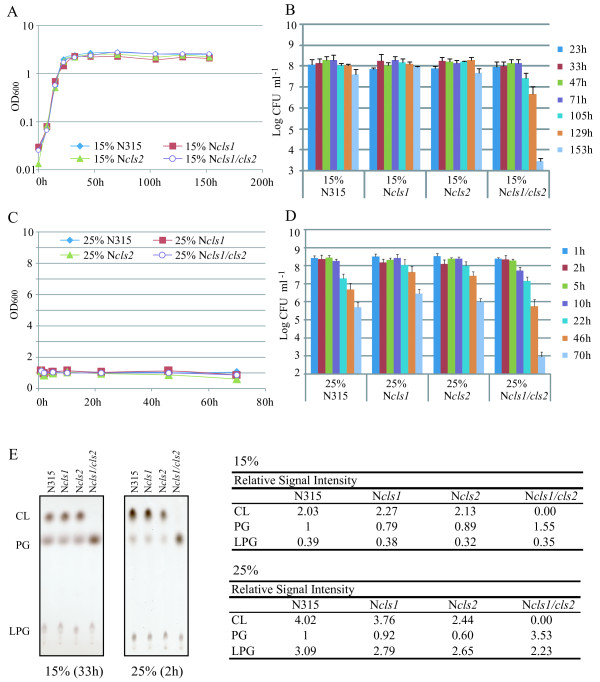
**Stationary-phase survival under high salinity**. Cells were grown in LB containing either 15% (**A**, **B**) or 25% (**C**, **D**) NaCl. **A**, **C**: ODs were measured at least twice, and the means are shown. **B**, **D**: The number of CFUs was determined at least three times. The means and standard deviations are shown. **E**: Thin-layer chromatography of phospholipids. Note that CL accumulated in the *cls2 *mutant. The relative signal intensities are shown on the right.

In a high-salinity medium (15% NaCl LB), the growth yield was reduced in N315 and *cls *mutants (Figure 6A), but the growth of *cls *mutants was not significantly different from that of the parent strain. In contrast, the number of *cls1/cls2 *double mutant CFUs was drastically reduced after ~105 to 153 h in high-salinity medium (Figure 6B). This reduction was not observed in either of the single mutants, suggesting that one *cls *gene is sufficient for long-term survival in high salinity. No growth was detected in medium containing 25% NaCl. Although the number of CFUs decreased gradually in both N315 and its *cls *mutants, the decrease was much faster for the *cls1/cls2 *double mutant after 46 h. Based on these findings, we conclude that CL is critical for staphylococcal fitness under conditions of high salinity.

No difference in susceptibility to antibiotics affecting cell walls (vancomycin, teicoplanin, cefarotin, cefmetazole, and cefazoline), quinolones (ofloxacin, norfloxacin, ciprofloxacin, and nalidixic acid), arbekacin, or the antimicrobial peptides ASABF-α [33] and nisin was observed between the N315 and its *cls *mutants (data not shown). The MIC of nisin for both *S*. *aureus *N315 and its *cls *mutants was 80 μg ml^-1^.

### Effect of *cls *mutations on L-form generation

**Figure 7:**
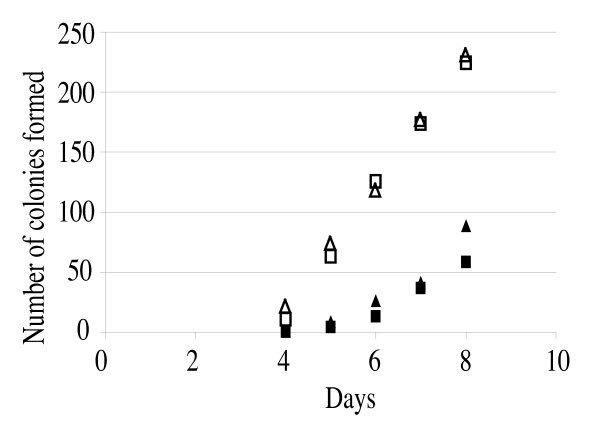
**L-form generation in MT01 and its *cls *mutants**. MT01: open squares; *cls1 *mutant: open triangles; *cls2 *mutant: filled squares; *cls1*/*cls2 *double mutant: filled triangles. L-forms are 'fried-egg-shaped' colonies that appear after prolonged incubation with cell-wall perturbing antimicrobials. The L-form has no cell wall, which we confirmed by disruption at low osmotic pressure. The means of at least two independent determinations are shown.

*Staphylococcus aureus *cannot form normal colonies in the presence of penicillin. After a prolonged incubation, colonies with a 'fried egg shape' emerge [34]. This adapted cell form is termed the L-form [35]. *Staphylococcus aureus *has especially high turgor pressure, and the L-form is induced under conditions of 5% NaCl and 5% sucrose. The L-form cell is able to grow without a cell wall, is Gram-negative, and lyses readily under hypotonic conditions (e.g., water). Thus, the L-form cell must have mechanisms allowing it to survive in such environments without the physical support of a cell wall. As one L-form strain has been shown to accumulate large amounts of CL [36], we investigated the possibility that CL is important in the generation of the L-form variant by constructing *cls *mutants in the MT01 strain, which is capable of generating the L-form. The lack of *cls *genes did not abolish L-form generation, although the efficiency of L-form generation was reduced in the *cls2 *single and *cls1/cls2 *double mutants, but not in the *cls1 *single mutant (Figure 7).

### Function of *cls1 *in stress responses

**Figure 8:**
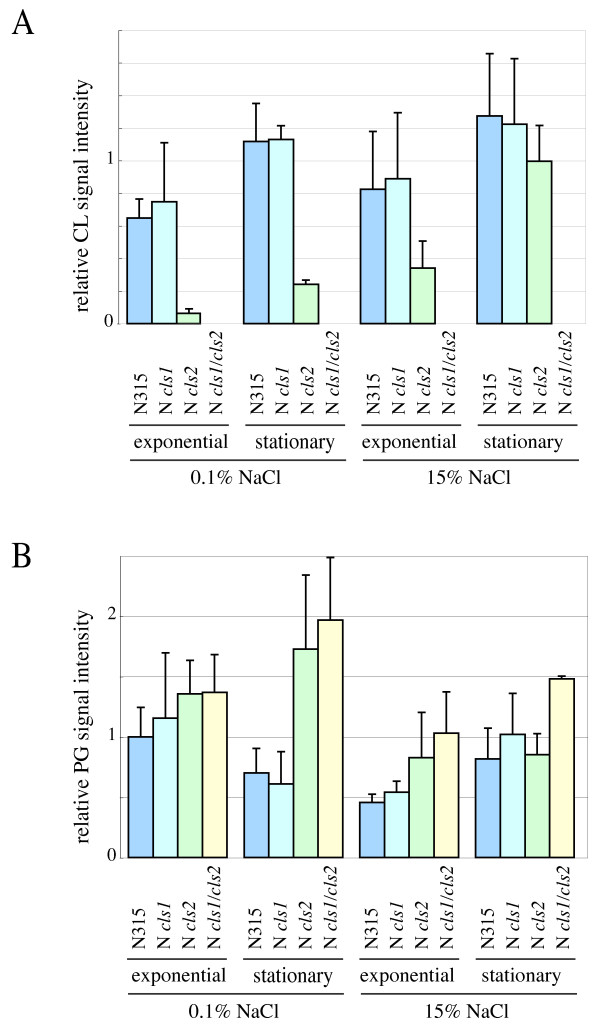
**Summary of the cardiolipin (CL) and phosphatidylglycerol (PG) signal intensities in each strain under distinct NaCl concentrations**. Strains cultured in LB containing 0.1% or 15% NaCl were harvested during exponential (3 h for 0.1% NaCl LB, 7 h for 15% NaCl LB) or stationary (23 h for 0.1% NaCl LB, 33 h for 15% NaCl LB) phase. The means and standard deviations of two independent determinations are shown. **A**: CL. **B**: PG.

**Figure 9:**
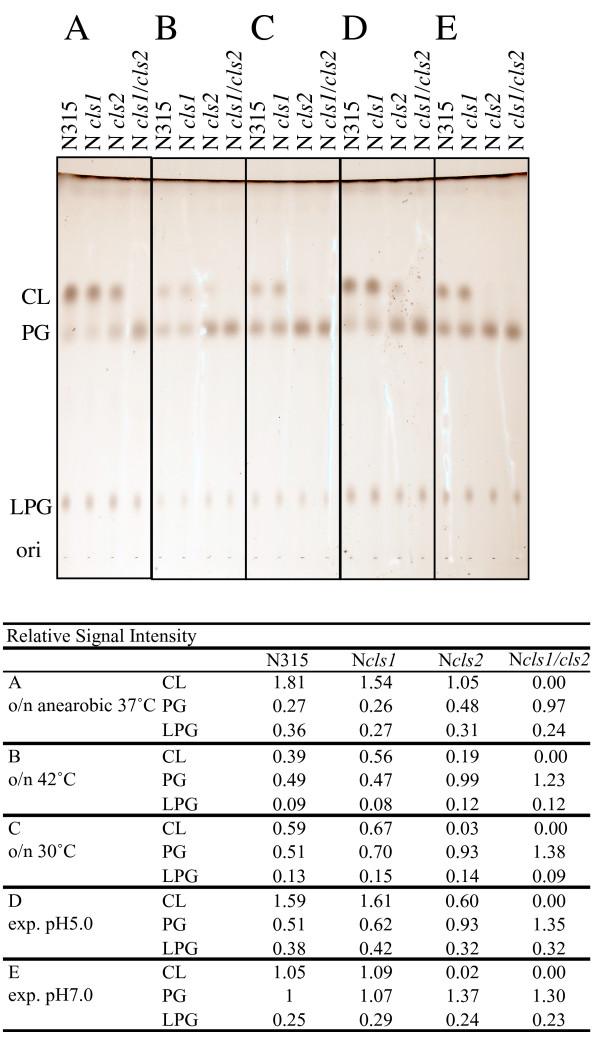
**Phospholipid analysis under defined conditions**. **A**: Anaerobic, 37°C, overnight culture (o/n); **B**: Aerobic, 42°C, o/n; **C**: Aerobic, 30°C, o/n; **D**: Aerobic, 37°C, pH 5, exponential-phase culture; **E**: Aerobic, 37°C, pH 7, exponential-phase culture. Relative signal intensities are shown at the bottom.

Figure 8 summarizes the CL accumulation in each strain grown under 0.1 and 15% NaCl concentrations. The total CL level did not differ detectably between the *cls1 *mutant and the parent strain grown in basal culture medium, indicating that under these conditions Cls2 is the dominant CL synthase. The *cls2 *mutant accumulated CL under high salinity, but not under low salinity. As the *cls1/cls2 *double mutant did not synthesize CL, the synthesis of CL by the *cls2 *mutant under high salinity must occur via Cls1. These synthesis profiles were shared among the mutant derivatives of N315 (Figure 8), 8325-4, and SH1000 (data not shown), suggesting that *S. aureus *Cls1 has a specific role under conditions of high salinity. We also tested the induction of Cls1-dependent CL accumulation in response to other stressors. Extreme conditions such as low pH, high temperature, or an anaerobic environment induced CL accumulation in the *cls2 *mutant (Figure 9).

## Discussion

Cardiolipin is known to play a role in the adaptive mechanisms of some bacteria to high salinity stress [15,20,37]. For example, a deficiency in CL decreases the growth rate in *B. subtilis *under conditions of 1.5 M (8.76%) NaCl [24]. Additionally, salt-sensitive *S. aureus *mutants contain no or only a small amount of CL [38,39]. Therefore, we were surprised to find that the growth of *S. aureus *under conditions of high salinity did not depend on CL (Figure 6). This may be attributable to the presence of other mechanisms, including species-specific systems such as variations in cell wall proteins [14], that give staphylococci the ability to cope with high-salt stress [11,40]. However, this study is, to our knowledge, the first to demonstrate that CL is important for long-term fitness of *S. aureus *under conditions of high salinity. This is an important finding in understanding the NaCl resistance of *S. aureus*, which is itself important for commensal growth on skin and mucus membranes, survival on dry surfaces during indirect transmission, and persistence in foods with a high salt content [41].

Cardiolipin depletion did not increase the susceptibility of *S. aureus *to cell wall-targeted antibiotics, suggesting that CL alone is not responsible for bacterial survival against these challenges. We also examined the susceptibility of *S. aureus *and its *cls *mutants to cationic antimicrobial peptides; because cardiolipin is negatively charged, a decrease in total negative charge on the membrane surface may contribute to reduced cationic antimicrobial peptide resistance [42]. However, CL depletion had no effect on susceptibility to the antimicrobial peptides ASABF-α and nisin. It is possible that the net negative charge is compensated for by other membrane components such as PG. In fact, the PG level was increased in the mutants that did not accumulate CL. The importance of positively charged lysyl-phosphatidylglycerol (LPG) (or MprF protein) in resistance to cationic antimicrobial peptides has been reported [43,44], and the LPG level was not different between wild-type *S*. *aureus *and *cls *mutants. In addition to the probable effect of cell surface charge, we have previously reported that cell wall thickness is an important factor affecting resistance to the antimicrobial peptide ASABF-α [33]. Furthermore, in the present study, ASABF-α-resistant strains had cell walls that interfered with CL extraction (Additional file 1, Figure S1). Cell wall thickness may also be related to resistance against other antimicrobial peptides in *S. aureus *[45,46].

Our data indicate that lysostaphin treatment is critical for the efficient extraction of CL from *S. aureus*. Previous reports have suggested that CL is not readily extractable from *B. subtilis *and other Gram-positive bacteria without lysozyme treatment [47]. This may be attributable to its large molecular mass (~1500 Da) relative to that of other phospholipids, owing to its four acyl residues. However, ~25-kDa globular hydrophilic molecules can pass freely through the ~2-nm holes in the peptidoglycan polymer that forms the cell wall of Gram-positive bacteria [48]. Instead, the efficiency of CL extraction is likely reduced by its physical interactions with cell wall components; for example, when CL is bound to cell wall components, it will not efficiently enter the organic phase during extraction.

The membrane of the L-form variants of *S. aureus *is thought to express certain features that support cell growth and survival in the absence of a rigid cell wall. One study reported that a particular L-form strain had an increased CL level [36]. Our data demonstrate that the *cls2 *gene is important for normal L-form generation. However, the *cls1/cls2 *double mutant still produced L-form cells, suggesting the existence of a CL-independent mechanism. Thus, multiple mechanisms may function in cooperation to generate L-form variants. The production of a number of factors such as carotenoids, catalase, coagulase, lipase, fibrinolysin, hemolysin, and enterotoxin changes upon L-form generation and reversion [49-52]. However, none of these represents a common L-form variant phenotype, suggesting that L-form generation is associated with a drastic phenotypic conversion. The increase in CL content may be important, but not essential, for membrane stabilization.

In this study, both *cls1 *and *cls2 *were shown to encode functional CL synthases. Under our experimental conditions, *cls2 *was the dominant CL synthase gene. The *cls1 *mutant did not differ from the parental strain in its growth rate, survival at stationary phase, total CL accumulation, or L-form generation. However, our data indicate that the synthesis of CL by Cls1 helps the *cls2 *mutant to survive prolonged incubation under high-salt conditions (Figure 5E), suggesting that Cls1 has a specific function under stress conditions if Cls2 is unavailable. Future studies should examine the functional characteristics of these two CL synthases, including possible differences in their subcellular localizations.

## Conclusions

Improved lipid extraction and molecular genetic analyses showed that both *cls1 *and *cls2 *participate in CL accumulation. The *cls2 *gene appears to serve a housekeeping function, while *cls1 *is active under stress conditions. *Staphylococcus aureus *can grow under conditions of high salinity without CL, but CL is required to survive prolonged high salinity stress and to generate L-form variants. This CL-dependent survival helps to explain the success of *S. aureus *as a human pathogen and skin/mucus membrane commensal.

## Methods

### Bacterial strains and culture conditions

The *S. aureus *strains used in this study are shown in Table 1. Luria-Bertani (LB) broth was the basic culture medium, and its NaCl content was modified as indicated. Cells were pre-cultured aerobically at 37°C overnight with shaking (180 rpm; BR-15; TAITEC, Tokyo, Japan). Culture inoculate (200 μl) was added to 40 ml of LB containing 0.1% NaCl or 15% NaCl in a 200 ml Erlenmeyer flask and incubated at 37°C with shaking (230 rpm; BR-23UM; TAITEC). To achieve the 25% NaCl culture condition, 0.4 ml of an overnight culture was mixed with 2 ml of LB containing 30% NaCl, and the culture was incubated at 37°C with shaking (180 rpm; BR-15; TAITEC). When necessary, the pH was adjusted to 7.0 or 4.8, and the cells were harvested at exponential phase before any change in pH. The growth rate was measured spectrophotometrically as optical density at 600 nm (OD_600_). Anaerobic growth was carried out at 37°C without shaking. Mutant isolation procedures used tryptic soy broth (TSB) or brain heart infusion (BHI) medium.

**Table 1:** Bacterial strains, plasmids, and primers used in this study

Strain or plasmid	Relevant characteristics	Source/reference
*S. aureus *strain		
RN4220	8325-4 derivative that accept foreign DNA	[55]
R *cls1*	RN4220 Δ*SA1155*; Cm^r^	This study
R *cls2*	RN4220 Δ*SA1891*; Tet^r^	This study
R *cls1*/*cls2*	RN4220 Δ*SA1155/*Δ*SA1891*; Cm^r ^Tet^r^	This study
N315	pre-methicillin resistant strain	[56]
N *cls1*	N315 Δ*SA1155*; Cm^r^	This study
N *cls2*	N315 Δ*SA1891*; Tet^r^	This study
N *cls1*/*cls2*	N315 Δ*SA1155/*Δ*SA1891*; Cm^r ^Tet^r^	This study
8325-4	NCTC 8325 cured of prophages, *rsbU*(-)	[57]
8 *cls1*	8325-4 Δ*SA1155*; Cm^r^	This study
8 *cls2*	8325-4 Δ*SA1891*; Tet^r^	This study
8 *cls1*/*cls2*	8325-4 Δ*SA1155/*Δ*SA1891*; Cm^r ^Tet^r^	This study
SH1000	8325-4 derivative, *rsbU *repaired, *rsbU*(+)	[58]
S *cls1*	SH1000 Δ*SA1155*; Cm^r^	This study
S *cls2*	SH1000 Δ*SA1891*; Tet^r^	This study
S *cls1*/*cls2*	SH1000 Δ*SA1155/*Δ*SA1891*; Cm^r ^Tet^r^	This study
MT01	isolate from healthy host, able to generate L-form	This study
MT01 *cls1*	MT01 Δ*SA1155*; Cm^r^	This study
MT01 *cls2*	MT01 Δ*SA1891*; Tet^r^	This study
MT01 *cls1*/*cls2*	MT01 Δ*SA1155/*Δ*SA1891*; Cm^r ^Tet^r^	This study
		
plasmids		
pMAD	thermosensitive, pE194^ts^-based delivery vector	[53]
pMADcat1155	pMAD-based *SA1155 *targeting vector	This study
pMADtet1891	pMAD-based *SA1891 *targeting vector	This study
pHY300PLK	shuttle vector, *tet *(Tet^R^)	Takara, Japan
pRIT5H	shuttle vector, *cm*(Cm^R^)	[54]
		
primers	5'-3'	
Tet-F(Sal)	CATATTGTCGACTAAGTGATGAAATACTG	
Tet-R(EcoRI)	GGAATTCCTGTTATAAAAAAAGGATCAAT	
CAT(Sal)F	CGAGTCGACGATAAAGTGGGATATTTT	
CAT(Eco)R	CGAATTCCGGGGCAGGTTAGTGACATT	
clsU1p	TGGATCCTGATATTGCTTACATACT	
clsU2p	TGGGTCGACAAAAAGTACAAATAGC	
clsD1p	CACAGATCTTATGGACTTTAGAAGTT	
clsD2p	TTTAGATCTCAATATCATCCAAATTA	
1891U1	CGGATCCAATAGTCCGACGATAGCT	
1891U2	GAAGTCGACGTCCTAATAGTAAGTA	
1891D1	TGAATTCACAAAAGCACGTTATGCT	
1891D2	TGAAGATCTAACATCACAACGGCATA	

Cm: chloramphenicol, Tet: tetracycline

### Lipid extraction and thin-layer chromatography

Cells equivalent to 8 × 10^8 ^CFU were collected at exponential or stationary phase, washed in 2% NaCl, and resuspended in 200 μl of 2% NaCl. Lysostaphin (0.1 mg ml^-1^) was added, and the mixture was incubated at 37°C for 3 min. The lysed cell suspension was extracted with chloroform-methanol. Briefly, a five-fold volume of chloroform-methanol (2:1; v/v) was added, mixed vigorously for 2 min, and left at room temperature for 10 min. Following the addition of a three-fold volume of chloroform-2% NaCl (1:1; v/v) and centrifugation, the lower layer was recovered and concentrated under vacuum. The lipids were dissolved in chloroform-methanol (2:1; v/v), applied to silica thin-layer chromatography (TLC) plates (Silica gel 60; Merck, Darmstadt, Germany), and developed with chloroform-methanol-acetic acid (65:25:10; v/v/v). The TLC plates were sprayed with CuSO_4 _(100 mg ml^-1^) containing 8% phosphoric acid and heated at 180°C to visualize the phospholipids. A digital image was obtained using a scanner, and signal intensities were quantified by ImageJ software (version 1.43U, NIH).

### Construction of mutants

To construct *S. aureus *mutants, we used pMAD, which is designed for efficient allelic replacement in Gram-positive bacteria [53], and incorporated the tetracycline or chloramphenicol resistance gene to make it suitable for use with erythromycin-resistant hosts such as *S. aureus *N315. The *tet *gene was amplified with primers Tet-F(Sal) and Tet-R(EcoRI) using pHY300PLK (Takara Shuzo Co. Ltd., Kyoto, Japan) as a template and ligated into the *Eco*RI-*Sal*I site of pMAD to generate pMADtet. The *cat *gene was amplified from pRIT5H [54] with primers CAT(Sal)F and CAT(Eco)R and ligated into the *Eco*RI-*Sal*I site of pMAD to generate pMADcat.

Target vectors were designed to replace the *SA1155 *(*cls1*) and *SA1891 *(*cls2*) genes with *cat *and *tet*, respectively. Two regions encompassing *SA1155 *were amplified with the primer pairs clsU1p and clsU2p (upstream region) and clsD1p and clsD2p (downstream region), restricted at the primer-attached sites, and sequentially ligated into the *Bam*HI-*Sal*I and *Bgl*II sites of pMADcat to generate the target plasmid pMADcat1155. Similarly, the upstream and downstream regions of *SA1891 *were amplified with the primer pairs 1891U1 and 1891U2, and 1891D1 and 1891D2, and then sequentially ligated into the *Bam*HI-*Sal*I and *Eco*RI-*Bgl*II sites of pMADtet to generate pMADtet1891. These target vectors were introduced into *S*. *aureus *RN4220 and N315 by electroporation. Each mutant was isolated as described previously [53]. Briefly, β-galactosidase-positive colonies carrying the target vector were plated on TSB agar (TSA) containing antibiotic (12.5 μg ml^-1 ^Cm or 5 μg ml^-1 ^Tet) and 100 μg ml^-1 ^X-gal, followed by incubation at 42°C overnight. Several resulting blue colonies were pooled and subjected to three cycles of growth in drug-free TSB at 30°C for 12 h and at 42°C for 12 h. Dilutions were plated on drug-free TSA plates containing 100 μg ml^-1 ^X-gal. Homologous recombination in white colonies was detected by PCR and Southern blot analyses. The *SA1155*/*SA1891 *double mutants of RN4220 and N315, the *SA1155 *and *SA1891 *single mutants, and the *SA1155*/*SA1891 *double mutants of SH1000, 8325-4, and MT01 were obtained by phage transduction. The absence of the genes in each mutant was confirmed by Southern blot analysis and/or PCR.

### Antibiotic and antimicrobial peptide susceptibility test

Cells were grown overnight in 5 ml of drug-free Muller-Hinton (MH) broth at 37°C with shaking (180 rpm; BR-1; TAITEC). These cells were diluted with MH (×10^-4^) and plated onto MH agar. Antibiotic susceptibilities of the strains were compared using the disk diffusion method (BD BBL sensi-Disk; Becton, Dickinson and Co., Franklin Lakes, NJ). The susceptibilities to ASABF-α were measured as described previously [33]. The minimum inhibitory concentration (MIC) of nisin (from *Lactococcus lactis*; Sigma, St. Louis, MO) was determined by microdilution with 10^4 ^cells per well and a 20-h incubation at 37°C.

### L-form induction

Cells were cultured in BHI without antibiotics, and 100 μl of the overnight culture were spread onto BHI agar plates containing 5% NaCl, 5% sucrose, 10% heat-inactivated horse serum, and 100 μg ml^-1 ^penicillin. The presence of serum selects for the stable L-form of *S. aureus *[34]. The plates were incubated at 37°C, and colonies showing the L-form ('fried egg shape') were counted for 8 days post-inoculation [34].

## Authors' contributions

MT carried out the phospholipid analyses and molecular genetic studies, and participated in manuscript preparation. RLO performed the high-salinity survival analyses, and YK performed the antimicrobial peptide susceptibility tests. SLT participated in the molecular genetic studies. YK, RLO, TO, and SS participated in designing the study. HH conceived of the study with KM and helped to coordinate the study. KM carried out molecular genetic studies, participated in the design and coordination of the study, and helped to draft the manuscript. All authors have read and approved the final manuscript.

## Supplementary Material

Additional file **Figure S1 - The phospholipid analysis of ASABF-α-susceptible strains and resistant strains**. Strains N315, NKSB, NKSBv, and MRSA no. 33 are susceptible to ASABF-α, and strains NKSBm, MRSA no. 7, and Mu50 are resistant [33]. Cells were harvested at stationary phase. Lipids were extracted by the chloroform-methanol method without (A) or with (B) the lysostaphin treatment. Solvent system: chloroform-methanol-acetic acid (65:25:10; v/v/v). Mu50 has unusually thick cell walls (ref*) and required higher lysostaphin concentration for efficient CL extraction (data not shown). ref*: Cui, L., X. Ma, K. Sato, K. Okuma, F. C. Tenover, E. M. Mamizuka, C. G. Gemmell, M. N. Kim, M. C. Ploy, N. El-Solh, V. Ferraz, and K. Hiramatsu. 2003. Cell wall thickening is a common feature of vancomycin resistance in *Staphylococcus aureus*. J Clin Microbiol 41:5-14. Click here for file
