# Supplementary figures and images for: Staphylococcus aureus requires cardiolipin for survival under conditions of high salinity
Source: BMC Microbiol. 2011 Jan 18;11:13. doi: 10.1186/1471-2180-11-13 (PMC3030509; doi:10.1186/1471-2180-11-13)

A

B

CL  
PGLPG  
oriN315  
NKSB  
NKSBm  
NKSBv  
No. 33  
No. 7  
Mu50N315  
NKSB  
NKSBm  
NKSBv  
No. 33  
No. 7  
Mu50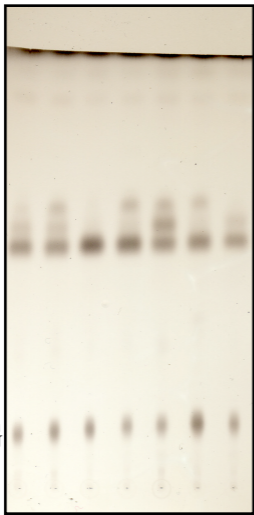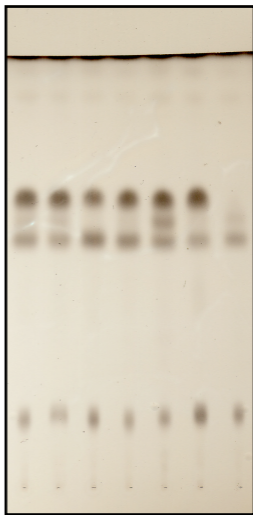

Supplement: Additional file — Figure S1 - The phospholipid analysis of ASABF-α-susceptible strains and resistant strains. Strains N315, NKSB, NKSBv, and MRSA no. 33 are susceptible to ASABF-α, and strains NKSBm, MRSA no. 7, and Mu50 are resistant [33]. Cells were harvested at stationary phase. Lipids were extracted by the chloroform-methanol method without (A) or with (B) the lysostaphin treatment. Solvent system: chloroform-methanol-acetic acid (65:25:10; v/v/v). Mu50 has unusually thick cell walls (ref*) and required higher lysostaphin concentration for efficient CL extraction (data not shown). ref*: Cui, L., X. Ma, K. Sato, K. Okuma, F. C. Tenover, E. M. Mamizuka, C. G. Gemmell, M. N. Kim, M. C. Ploy, N. El-Solh, V. Ferraz, and K. Hiramatsu. 2003. Cell wall thickening is a common feature of vancomycin resistance in Staphylococcus aureus. J Clin Microbiol 41:5-14. [file 1471-2180-11-13-S1.PDF]
